# Effects of methanolic extract of *Cuminum cyminum* on total serum cholesterol in ovariectomized rats

**DOI:** 10.4103/0253-7613.51353

**Published:** 2009-04

**Authors:** Sarika S. Shirke, Aarti G. Jagtap

**Affiliations:** Department of Pharmacology, Bombay College of Pharmacy, Kalina, Santacruz (E), Mumbai - 400 098, India. E-mail: dragjagtap@gmail.com

Sir,

Estrogens have a protective role in lipid metabolism[1] and postmenopausal women are at high risk for developing cardiovascular disorders due to estrogen deficiency. As suggested by many epidemiological studies, hormone replacement therapy (HRT) in postmenopausal women appears to be associated with reduction in the risks of coronary heart disease.[2] Protection against cardiovascular disorders is an attractive side of HRT in postmenopausal women, but at the same time it is associated with serious side effects like breast and endometrial cancers and is also associated with patient incompatibility which presents many limitations in long-term use.

Phytoestrogens, polyphenolic non-steroidal plant compounds with estrogen-like biological activity are currently offering an effective and safe alternative to hormone replacement therapy. Owing to the estrogen-like property they are effective in a variety of aliments such as menopausal symptoms and postmenopausal disorders, such as osteoporosis and cardiovascular disorders.[3] Ample evidence from epidemiological studies and clinical trials suggests the plausibility of a causal, inverse relationship between phytoestrogens and cardiovascular disease. The well established low rates of cardiovascular disease and high intake of dietary phytoestrogens in Asian populations are consistent with a potential cardioprotective effect of phytoestrogens.[2]

Fruits of *Cuminum cyminum* (Apiaceae), commonly known as *jeera* are consumed as condiment across the globe. Fruits of *Cuminum cyminum* (CC) are rich in estrogenic isoflavonoids luteolin and apigenin.[4] CC extract is included as one of the major components in some polyherbal formulations because of its estrogenic nature. The estrogenic activity of acetone extract of CC has been reported earlier in immature ovariectomized rats.[5] It has been reported to reduce plasma cholesterol levels in diabetic rats.[6]

The present study was undertaken to evaluate hypocholesterolemic effect of methanolic extract of *Cuminum cyminum* (MCC) as a part of anti-osteoporotic evaluation of MCC in ovariectomized (OVX) rats. Methanol was the solvent of choice owing to its excellent penetration and extraction ability.

Forty adult Sprague Dawley female rats of 3 months age, weighing 200-250 gm were divided into four groups with N = 10 per group. Three groups were bilaterally ovariectomized while one was sham operated (SH control). MCC 1000 mg/kg and estradiol benzoate equivalent to 0.15 mg/kg of estradiol were administered to OVX rats per orally for 10 weeks. MCC was administered in two divided doses with 2 ml/kg dose volume. SH control and OVX control groups were administered with vehicle 0.5% sodium carboxy methyl cellulose. The treatment was started the day after ovariectomy.

At the termination of study, blood was collected from overnight fasted animals; serum was harvested and preserved at 4°C until the analysis. Total serum cholesterol was determined colorimetrically using standard cholesterol estimation kit (Erba). Results were analyzed by one-way ANOVA at *P* < 0.05.

OVX rats showed significantly elevated levels of serum total cholesterol at the end of 10 weeks as compared to SH control. Treatment with estradiol and MCC both significantly decreased total cholesterol levels in serum. The decrease in total serum cholesterol caused by MCC was significantly greater than that caused by standard drug estradiol. It was observed that total cholesterol levels in MCC treated rats were lower but not significantly than SH control rats. The results indicated that estradiol as well as MCC protected OVX rats against increased cholesterol levels due to ovariectomy while MCC was better than estradiol [Table 1].

**Table 1:** Effects of oral treatment of estradiol and MCC (methanolic extract of *Cuminum cyminum*) on total serum cholesterol in ovariectomized (OVX) rats

*Treatment*	*Total serum cholesterol (mg/dl)*
Sham control	76.68 ± 4.14
OVX control	107.92# ± 11.76
Estradiol (0.15 mg/kg)	82.14* ± 3.47
MCC (1000 mg/kg)	73.39*;α ± 1.08

Values are Mean ± SEM (N = 10);

# : Significantly different at *P* < 0.05 vs. Sham control;

* : Significantly different at *P* < 0.05 vs. OVX control and α: Significantly different at *P* < 0.05 vs. Estradiol.

It is likely that the hypocholesterolemic effect of MCC may be due to its phytoestrogenic content but to validate the claims comprehensive and detailed investigation has to be undertaken.

Thus methanolic extract of *Cuminum cyminum* can be a potential candidate to be explored for the treatment of menopausal disorders, especially cardiovascular disorders in postmenopausal women.
